# The Kabachnik–Fields Reaction: Mechanism and Synthetic Use

**DOI:** 10.3390/molecules171112821

**Published:** 2012-11-01

**Authors:** György Keglevich, Erika Bálint

**Affiliations:** 1 Department of Organic Chemistry and Technology, Budapest University of Technology and Economics, 1521 Budapest, Hungary; 2 Research Group of the Hungarian Academy of Sciences, Department of Organic Chemistry and Technology, Budapest University of Technology and Economics, 1521 Budapest, Hungary

**Keywords:** Kabachnik–Fields reaction, α-aminophosphonates, reaction pathway, environmentally friendly, microwave, solventless

## Abstract

The Kabachnik–Fields (phospha-Mannich) reaction involving the condensation of primary or secondary amines, oxo compounds (aldehydes and ketones) and >P(O)H species, especially dialkyl phosphites, represents a good choice for the synthesis of α-aminophosphonates that are of significant importance due to their biological activity. In general, these three-component reactions may take place via an imine or an α-hydroxy-phosphonate intermediate. The monitoring of a few Kabachnik–Fields reactions by *in situ* Fourier transform IR spectroscopy has indicated the involvement of the imine intermediate that was also justified by theoretical calculations. The Kabachnik–Fields reaction was extended to >P(O)H species, comprising cyclic phosphites, acyclic and cyclic *H*-phosphinates, as well as secondary phosphine oxides. On the other hand, heterocyclic amines were also used to prepare new α-amino phosphonic, phosphinic and phosphine oxide derivatives. In most cases, the synthesis under solvent-free microwave (MW) conditions is the method of choice. It was proved that, in the cases studied by us, there was no need for the use of any catalyst. Moreover, it can be said that sophisticated and environmentally unfriendly catalysts suggested are completely unnecessary under MW conditions. Finally, the double Kabachnik–Fields reaction has made available bis(phosphonomethyl)amines, bis(phosphinoxidomethyl)amines and related species. The bis(phosphinoxidomethyl)amines serve as precursors for bisphosphines that furnish ring platinum complexes on reaction with dichlorodibenzonitriloplatinum.

## 1. Introduction

**Scheme 1:**
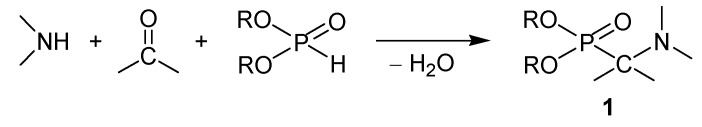
General scheme for the Kabachnik–Fields reaction.

The basic method for the preparation of α-aminophosphonates, valuable synthetic equivalents and biologically active substrates, involves the condensation of a primary or secondary amine, a carbonyl compound (aldehyde or ketone) and dialkyl phosphite (Scheme 1) [1,2]. 

α-Aminophosphonic acids, considered as phosphorus analogues of α-amino acids, have attracted much attention in drug research due to their low mammalian toxicity. They are important targets in the development of antibiotics, antiviral species, antihypertensives, and antitumour agents based on their effect as inhibitors of GABA-receptors, enzyme inhibitors and anti-metabolites [3,4,5,6,7,8,9]. Diaryl α-amino-phosphonate derivatives are selective and highly potent inhibitors of serine proteases, and thus can mediate the patho-physical processes of cancer growth, metastasis, osteoarthritis or heart failure [10]. Dialkylglycine decarboxylase [9] and leucine aminopeptidase [11] are also inhibited by α-amino-phosphonates. Cyanoacrylate [12] and amide derivatives [13] of α-aminophosphonates are active antiviral compounds and inactivators of the tobacco mosaic virus. Certain α-aminophosphonates were proved to be suitable for the design of continuous drug release devices due to their ability to increase the membrane permeability of a hydrophilic probe molecule [14].

## 2. Possible Pathways for the Kabachnik–Fields Reaction

**Scheme 2:**
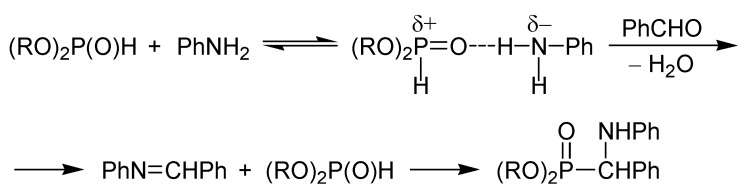
The “imine” mechanism proposed for a Kabachnik–Fields reaction [15,16].

Cherkasov *et al*. studied the mechanism of the Kabachnik–Fields reaction in detail. One possibility is that an imine (a Schiff base) is formed from the carbonyl compound and the (primary) amine, and then the dialkyl phosphite is added on the C=N unit of the intermediate. The other route assumes the formation of an α-hydroxyphosphonate by the addition of the dialkylphosphite to the carbonyl group of the oxo component, then the hydroxyphosphonate undergoes substitution by the amine to furnish the α-aminophosphonate. On the basis of kinetic studies, it was concluded that the mechanism is dependent on the nature of the reactants. For example, the condensation of aniline, benzaldehyde and a dialkyl phosphite was assumed to follow the “imine” mechanism. Interestingly it was found that before the condensation of the aniline and the benzaldehyde, an H-bond is formed between the P=*O* function of the phosphite and the *H*N unit of the amine (Scheme 2) [15,16].

**Scheme 3:**
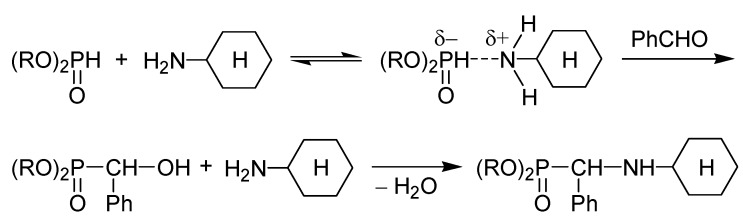
The “α-hydroxyphosphonate” mechanism proposed for a Kabachnik–Fields reaction [15,17].

In another case, Cherkasov *et al*. suggested that the reaction of the more nucleophilic cyclohexyl-amine, benzaldehyde and a dialkyl phosphite takes place via the “hydroxyphosphonate” route. Here again an interaction was substantiated to precede the addition of the dialkylphosphite on the C=O unit of the oxo-compound. According to this, an H-bond is formed between the P(O)*H* moiety of the phosphite and the nitrogen atom of the amine (Scheme 3) [15,17].

Later, however, Zefirov and Matveeva proved that the condensation of cyclohexylamine, benzaldehyde and dialkyl phosphite follows the “imine route”, and concluded that there is no real experimental evidence for the hydroxyphosphonate route [18]. It is also worth mentioning that the reaction of cyclohexylamine, benzaldehyde and dibutylphosphine oxide, that may be regarded as an extended Kabachnik–Fields condensation, was shown to proceed according to the “imine” mechanism [15,19]. It seems probable that the actual mechanism is dependent on the components of the reaction, although the “imine” route seems to be more general than the route involving an “α-hydroxy-phosphonate” intermediate [3]. R. Gancarz and I. Gancarz substantiated that a reversible formation of the α-hydroxyphosphonate may also occur, and if it is rearranged to the corresponding phosphate, this becomes a “dead-end” route [20]. It can be said that in the Kabachnik–Fields reaction, a soft nucleophile (the dialkyl phosphite) and a hard nucleophile (the amine) compete for the electrophilic carbonyl compound. The softer the carbonyl compound is, the faster it reacts with the softer P-nucleophile and the slower it reacts with the harder amine nucleophile [21].

**Scheme 4:**
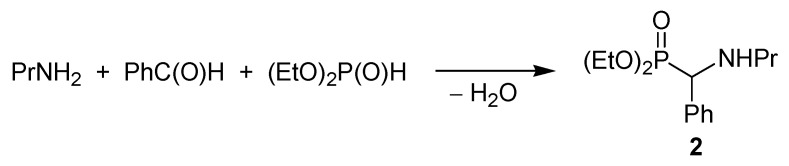
The Kabachnik–Fields reaction studied by us.

We wished to investigate the phospha-Mannich condensation of *n*-propylamine, benzaldehyde and diethyl phosphite (Scheme 4) by following the reaction utilizing *in situ* Fourier transform (FT) Infra Red (IR) spectroscopy [22].

**Scheme 5:**
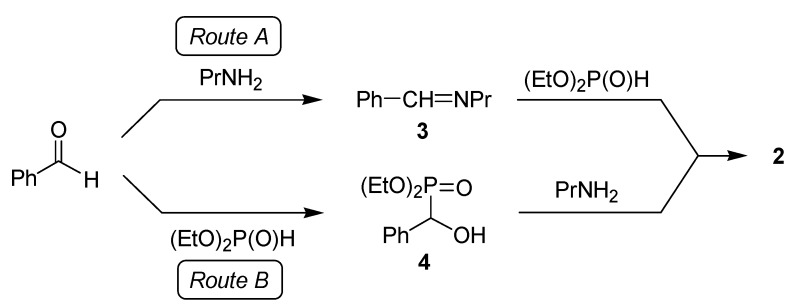
Possible routes for the Kabachnik–Fields reaction studied by us.

The possible reaction paths are shown in Scheme 5. The question was whether the imine **3** or the α-hydroxyphosphonate **4** is the intermediate during the formation of the corresponding α-aminophosphonate **2**.

**Figure 1:**
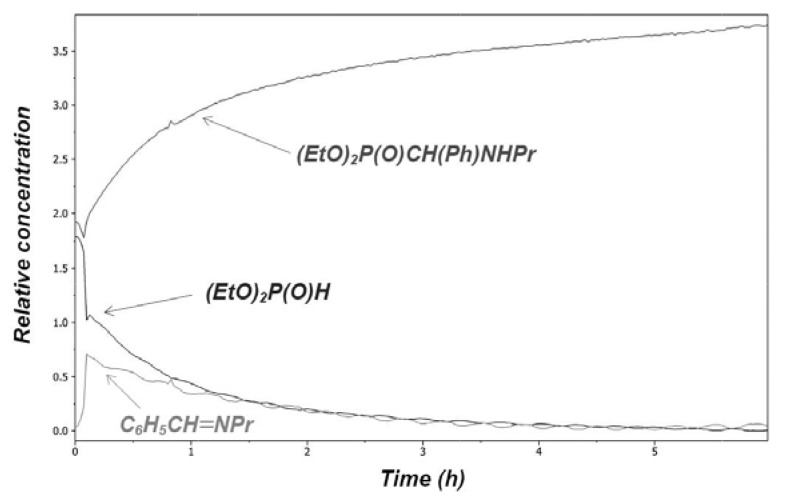
Concentration profile for the Kabachnik–Fields reaction studied at 80 °C in acetonitrile.

The reaction carried out at 80 °C in acetonitrile was monitored by registering a 3D IR diagram. On the basis of the characteristic ν_C=N_ stretching vibration at 1,648 cm^–1^, the imine **3** could be observed as a transient species. It was possible to obtain a relative concentration—time diagram for the components (Figure 1) by deconvolution of the 3D IR diagram. It can be seen that the imine intermediate **3** reaches its maximum concentration after a 10 min reaction time [22].

**Scheme 6:**
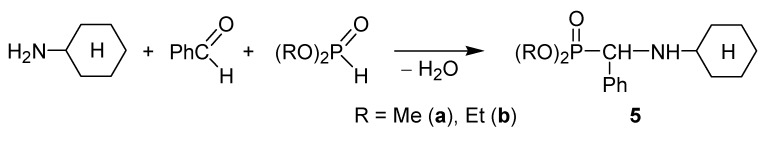
Another Kabachnik–Fields reaction investigated by us.

It was shown above that there was also controversy over the mechanism of the Kabachnik–Fields condensation of cyclohexylamine, benzaldehyde and dialkyl phosphites (Scheme 6) [15,17,18]. We sought to clarify the situation by *in situ* FT IR spectroscopy [23].

**Scheme 7:**
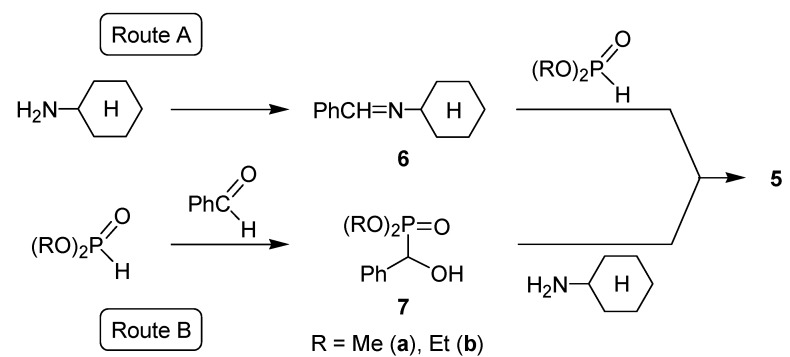
Possible pathways for the second model investigated.

**Figure 2:**
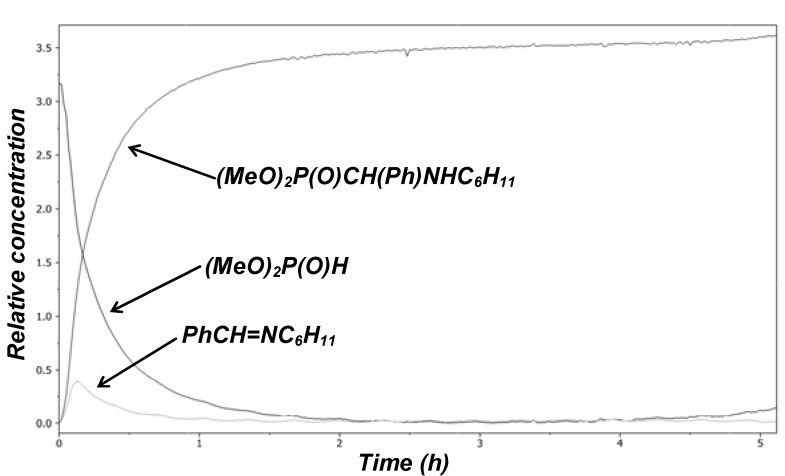
Concentration profile for the Kabachnik–Fields reaction studied at 80 °C in acetonitrile.

From among the two possible intermediates **6** and **7**, again the imine **6a** could be detected on the basis of the ν_C=N_ = 1,644 cm^–1^ absorption as the transient species for α-aminophosphonate **5a** (Scheme 7). The intermediacy of imine **6** can be seen in Figure 2.

Relative energies for the possible intermediates **6** and **7** and for α-aminophosphonate **5** were calculated by the B3LYP/6-31G** method and then refined by the the B3LYP/6-311G**^++^method provided that dimethyl phosphite is the reactant. It can be seen from Table 1 that the formation of the imine **6** goes with significantly lower energy gain than that of the α-hydroxyphosphonate **7**. On the one hand, the imine **6** would like to be stabilized further by reaction with the dimethyl phosphite on way to the α-aminophosphonate **5**. On the other hand, the hydroxyphosphonate **7** is too stable to react further to the aminophosphonate **5**. The conversion of **7** to **5** represents only a slight energy gain of 2.4 kJ/mol. In other words, there is no significant driving force for the substitution [23].

**Table 1:** Relative energies for the four states calculated.

Species	Relative energy (kJ/mol)
Reactants (benzaldehyde, cyclohexylamine and dimethyl phosphite)	0.0
Imine intermediate **6**	–18.6
α-Hydroxyphosphonate intermediate **7**	–40.5
Product **5**	–42.9

## 3. Microwave-Assisted Solvent- and Catalyst-Free Approach for the Synthesis of α-Amino-phosphonates and Related Derivatives

**Scheme 8:**
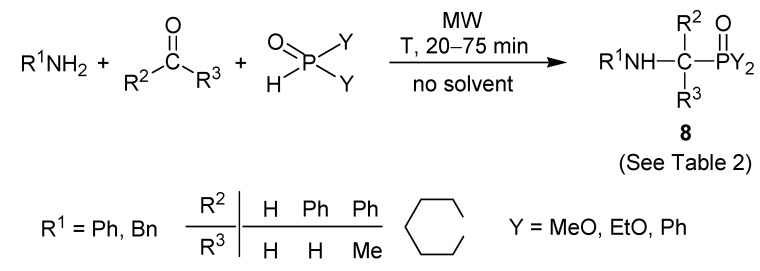
General scheme for the solventless, catalyst-free MW-assisted Kabachnik–Fields reactions studied.

Although a lot of catalytic variations to carry out three-component Kabachnik–Fields condensations have been described, we found that the most straightforward synthesis is when the reactants are irradiated with microwave (MW) in the absence of any catalyst or solvent. The solventless and MW-assisted approach was useful in the synthesis of a few α-aminomethylphosphonates [24]. We used aniline or benzylamine as the amine, formaldehyde, benzaldehyde, acetophenone and cyclohexanone as the oxo-component and dialkyl phosphites and diphenylphosphine oxide as the >P(O)H reactant. The α-aminophosphonates and α-aminophosphine oxide products are represented by structure **8** in general Scheme 8 [25]. The detailed results are listed in Table 2. The comparative results of the catalytic versions were also included. A detailed account on the conditions of the catalytic reactions is provided in Table 3. In a part of the cases, such as in the example covered by reference [26], the catalytic versions could already be carried out at room temperature.

**Table 2:** Kabachnik–Fields reactions carried out without the use of a solvent and a catalyst under MW irradiation [25].

Entry	R^1^	R^2^	R^3^	Y	Product	T (°C)	Yield (%)	Yield (%) of catalytic methods [ref.] ^†^
1	Ph	H	H	EtO	**8a**	80 ^a^	91	
					****	100 ^b^		
2	Ph	H	H	MeO	**8b**	80 ^a^	80	
						80 ^b^		
3	Ph	H	H	Ph	**8c**	80	94	
4	Bn	H	H	EtO	**8d**	100	81	
5	Bn	H	H	Ph	**8e**	80	88	
6	Ph	H	Ph	EtO	**8f**	100	93	98 [27], 85 [28], ~95 [29], 88 [30], 79 [31], 93 [32], 92 [33], ~90 [34], 96 [35], 60 [36], 86 [37], 92 [38]
7	Ph	H	Ph	MeO	**8g**	100	86	98 [24], 98 [27], 92 [33]
8	Ph	H	Ph	Ph	**8h**	80	87	
9	Bn	H	Ph	EtO	**8i**	100	83	99 [28], 84 [29], 92 [30], 85 [31], 91 [32], 91 [33], 92 [35], 92 [36], 93 [38]
10	Bn	H	Ph	MeO	**8j**	100	87	95 [27], 82 [33]
11	Ph	Me	Ph	EtO	**8k**	120	80	75 [27], 74 [30], 63 [35], 18 [36]
12	Bn	Me	Ph	EtO	**8l**	120	84	92 [26], 80 [27], 81 [38]
13	Bn	Me	Ph	Ph	**8m**	100 ^a^	80	
						120 ^b^	80	
14	Ph	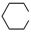		EtO	**8n**	120	81	92 [27], ~72 [29], 86 [30], 47 [31], 87 [37]
15	Bn	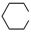		EtO	**8o**	120	91	85 [26], 90 [27], 83 [31], 80 [33], 85 [38]
16	Bn	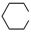		MeO	**8p**	120	85	92 [27]
17	Bn	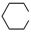		Ph	**8q**	100 ^a^	80	
						120 ^b^		

^†^ for details see Table 3; ^a^ condensation of the oxo-component and the amine; ^b^ addition of the >P(O)H species to the Schiff-base.

**Table 3:** Kabachnik–Fields reactions carried out in the presence of catalysts.

Catalyst	Solvent	MW/Δ	T [°C]	t	Yield (Product) [%]	Ref.
Phthalocyanine-AlCl	CH_2_Cl_2_	–	26 ^a^	12 h	92 (**8b**), 85 (**8p**)	[26]
Mg(ClO_4_)_2_	–	–	26	2 min/8 h	90–98 ( **8f**, **8g**, **8j**, **8n-p**)	[27]
Mg(ClO_4_)_2_	–	Δ	50-80	45 min–12 h	80–99 (**8f**, **8i**, **8l**,**8n-p**)	[27,28]
Mg(ClO_4_)_2_	EtOH	Δ	50	5 h/12 h	85 (**8f**), 99 (**8i**)	[28]
M(OTf)_n_ M = Li, Mg, Al, Cu, Ce	–	Δ	80	20 min–3.5 h	72–95 (**8f**, **8i**, **8n**)	[29]
GaI_3_	CH_2_Cl_2_	–	26	3–6 h	74–92 (**8f**, **8i**, **8k**, **8n**)	[30]
In(OTf)_3_	THF	Δ	66	21–35 h	47–85 (**8f**, **8i**, **8n**, **8o**)	[31]
BiNO_3_	–	– ^b^	26	10 h	93 (**8f**), 91 (**8i**)	[32]
BiCl_3_	MeCN	Δ	80	6–15 h	80–92 (**8f**, **8g**, **8i**, **8j**, **8o**)	[33]
FeCl_3_	EtOH (or solvent free)	–	26		~90 (**8f**)	[34]
YbCl_3_	MeCN	–	26	24 h	63–96 (**8f**, **8i**, **8k**)	[35]
SmI_2_ (+ 4 Å mol sieves)	MeCN	Δ	80	24 h	18–92 (**8f**, **8i**, **8k**)	[36]
ceric ammonium nitrate	MeCN	–	26	3 h	86 (**8f**), 87 (**8n**)	[37]
InCl_3_	THF	Δ	66	9–12 h	81–93 (**8f**, **8i**, **8l**, **8o**)	[38]
InCl_3_	DMF	MW	no data	2 min	82 (**8f**) ^c^	[39]
InCl_3_	[bmim][PF_6_]	MW	no data	2 min	91 (**8f**) ^c^	[39]
Ln(OTf)_3_ Ln = Yb, Sc, Dy, Sm	DMF	MW	no data	2 min	72 ( **8f**) ^c^	[39]
Ln(OTf)_3_ Ln = Yb, Sc, Dy, Sm	[bmim][PF_6_]	–	26	27 h	92 (**8f**) ^c^	[40]
Ln(OTf)_3_ Ln = Yb, Sc, Dy, Gd	[bmim][PF_6_]	MW	no data	2 min	89 (**8f**) ^c^	[39]
the solvent acts as catalyst	[bmim][BF_4_]	–	26	5 h/8 h	90 (**8f**), 84 (**8f**)	[41]

^a^ Diethyl phosphite was added to preformed imines; ^b^ Was also performed under MW; ^c^ The product was extracted with benzene.

**Scheme 9:**
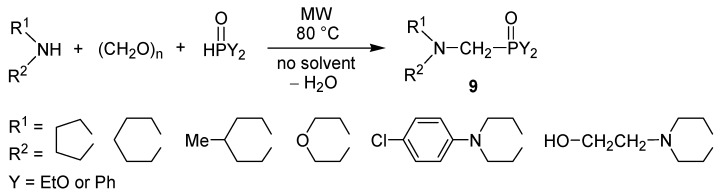
Kabachnik–Fields reactions applying *N*-heterocycles as the amine component.

On the basis of our experimental data, there is no need to use exotic (expensive and environmentally unfriendly) catalysts. In most cases, the solvent- and catalyst-free MW-assisted reactions give excellent results. Further exploration of catalysts does not seem to be justified in this field. Next, our method was extended to phospha-Mannich condensations involving heterocyclic amines (Scheme 9) [42]. Diphenylphosphine oxide was also used as the P-component.

**Scheme 10:**
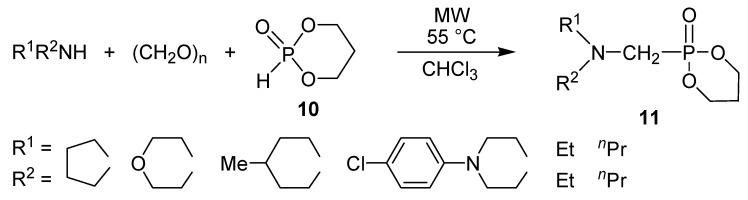
Kabachnik-Fields reactions applying 1,3,2-dioxaphosphorine oxide as the P-reactant.

In another series of reactions, 1,3,2-dioxaphosphorine oxide (**10**) was used as the phosphite (Scheme 10) [43]. In this way “double” heterocyclic derivatives were prepared. These reactions were more efficient in the presence of a solvent.

**Scheme 11:**
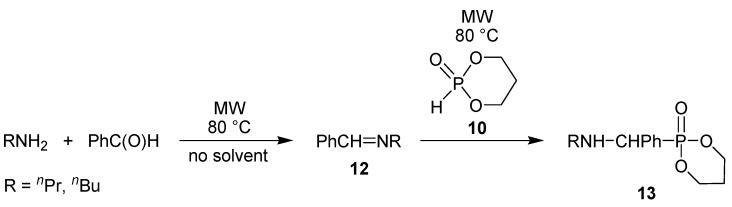
Synthesis of α-aminophosphonates via the imine intermediate.

Applying 1,3,2-dioxaphosphorine oxide (**10**) together with benzaldehyde, the steric hindrance prevented the efficient condensation. It was better to prepare the imine **12** first and to react it separately with the cyclic phosphite **10** (Scheme 11) [43].

**Scheme 12:**
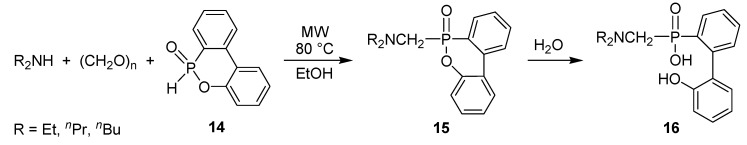
Kabachnik–Fields reaction applying a dibenzooxaphosphorine oxide as the P-reactant.

Dibenzo[*c.e*][1,2]oxaphosphorine (**14**) was also utilized in the synthesis of P-heterocyclic derivatives. In this case, the primarily formed product **15** underwent opening of the hetero ring by reaction with the water formed (Scheme 12) [43].

**Scheme 13:**
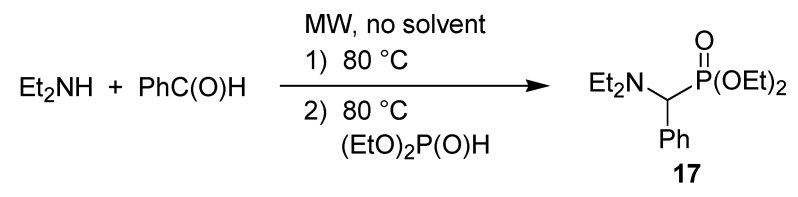
Synthesis of an α-aminophosphonates in two steps.

For the preparation of diethyl α-diethylaminophenylmethylphosphonate (**17**), the two-step approach led to better results. The aldehyde–amine adduct formed primarily was reacted with diethyl phosphite to afford product **17** (Scheme 13) [43].

**Scheme 14:**
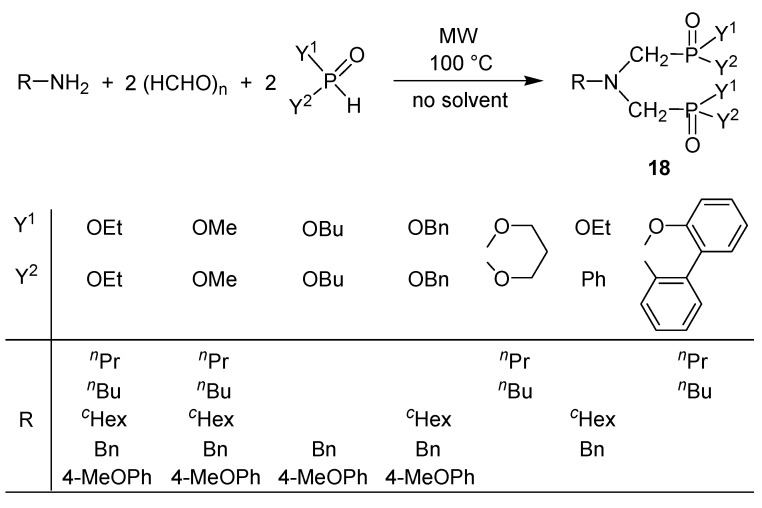
Synthesis of bis(phosphonomethyl)amines and related derivatives by the double Kabachnik–Fields reaction.

**Scheme 15:**
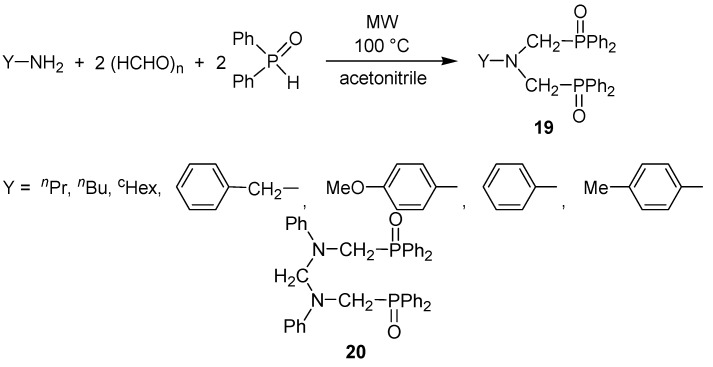
Bis(phosphinoxidomethyl)amines by the double phospha-Mannich reaction.

The MW-assisted solventless procedure was useful in the synthesis of a series of bis(phosphonomethyl)amines and related derivatives marked as **18** (Scheme 14) [44,45,46]. Product **18** could be obtained in yields, mostly above 80%. The double Kabachnik-Fields reaction was then extended to the synthesis of bis(phosphinoxidomethyl)amines **19**. In these cases, heterogenity of the reaction mixture requested the use of a solvent that was acetonitrile (Scheme 15) [44,45,46]. The use of aniline as the amine component led to by-product **20** besides the expected product **19** (Y = Ph) [46].

**Scheme 16:**
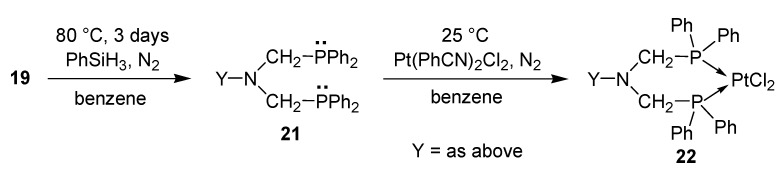
Synthesis of ring platinum complexes from bis(phosphinoxidomethyl)amines.

The bis(phosphinoxidomethyl)amines **19** served as precursors for bis(phosphinomethyl)amine bidentate P-ligands **21** by double deoxygenation. The bisphosphines **21** so formed were reacted with half an equivalent of dichlorodibenzonitriloplatinum to furnish ring platinum complexes **22** (Scheme 16) [45,46].

**Scheme 17:**
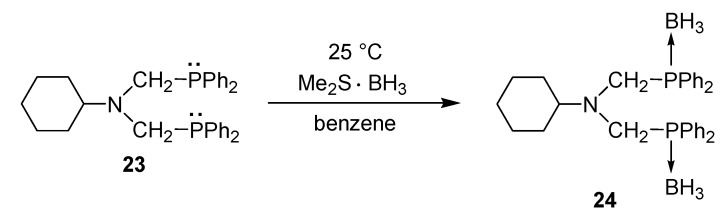
Stabilization of a bis(phosphinomethyl)amine as a bis(borane complex).

The bidentate P-ligands may be stored as their phosphine-borane complexes. This is shown in the example of the **23** → **24** conversion (Scheme 17). In general, the phosphine can be regenerated from the phosphine-borane by heating with a secondary amine, such as diethylamine, in an aromatic solvent [47].

**Scheme 18:**
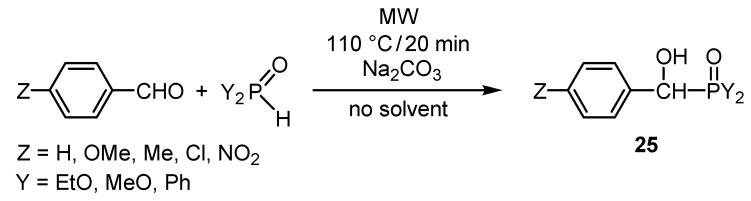
MW-assisted synthesis of α-hydroxyphosphonates and α-hydroxyphosphine oxides.

The MW-assisted catalytic addition of dialkyl phosphites on the carbonyl group of a series of benzaldehyde derivatives was also elaborated (Scheme 18) [48]. The α-hydroxyphosphonates (**25**, Y = RO) are potential intermediates of the Kabachnik–Fields reaction. The use of diphenylphosphine oxide in the addition led to the formation of α-hydroxyphosphine oxides (**25**, Y = Ph).

**Scheme 19:**
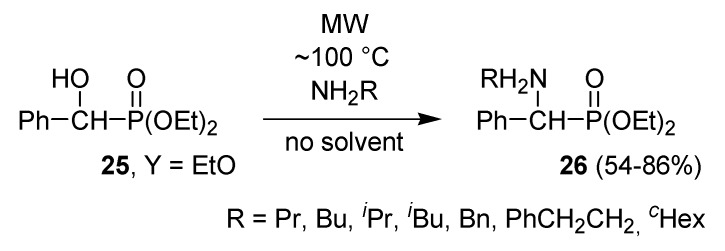
Preparation of α-aminophosphonates by substitution of α-hydroxyphosphonates.

The addition of dialkyl phosphites to α-ketophosphonates led to 1-hydroxymethylene-bisphosphonates [49,50]. It was interesting to find that, as a consequence of the neighboring group effect of the P=O moiety, α-hydroxyphosphonate **25** (Y = EtO) could be readily converted to the corresponding α-aminophosphonates (**26**) (Scheme 19) [51]. Quantum chemical calculations justified the beneficial neighboring group effect of the P=O moiety [51].

## 4. Conclusions

In conclusion, recent results obtained in the study of the Kabachnik–Fields reaction have been summarized. This mini-review sheds light on the new developments regarding mechanistic and synthetic aspects showing that the phospha-Mannich reaction remains an evergreen topic for organic chemists. On the one hand, the mechanism of the Kabachnik–Fields reaction still reserves some surprises, on the other hand, the 3-component condensation is an ideal subject for green chemical reactions. In addition, the α-aminophosphonate and α-aminophosphine oxide products are biologically active substrates.
